# Effects of High-concentration contrast material and low-voltage CT on contrast for multiphasic CT of the upper abdomen: comparison using the simulation with virtual monochromatic imaging obtained by fast-switch kVp dual-energy CT

**DOI:** 10.1186/2193-1801-3-234

**Published:** 2014-05-08

**Authors:** Makoto Sakane, Tonsok Kim, Masatoshi Hori, Hiromitsu Onishi, Atsushi Nakamoto, Takahiro Tsuboyama, Mitsuaki Tatsumi, Noriyuki Tomiyama

**Affiliations:** Department of Radiology, Osaka University Graduate School of Medicine, D1, 2-2, Yamadaoka, Suita, Osaka, 565-0871 Japan

**Keywords:** High-concentration contrast material, Low-voltage CT, Fast-switch kVp Dual-energy CT, Virtual monochromatic imaging

## Abstract

**Objective:**

The purpose of this study was to compare the effects of high-concentration contrast material and low-voltage CT simulated by virtual monochromatic (VM) imaging on contrast enhancement at multiphasic CT of the upper abdomen.

**Methods:**

This study included 72 patients who underwent CT during early arterial (EAP), late arterial and portal venous phases after 300-mgI/ml (Group A; 34 patients) or 350-mg/ml (Group B; 38 patients) contrast-material injection at the same volumetric rate (0.067 mL/sec/kg). VM images were generated at 50 and 65 keV. Contrast-to-noise ratios (CNRs) of aorta, portal vein, and liver parenchyma were calculated and statistically compared.

**Results:**

Mean CNRs for 50-keV VM images were significantly higher than 65-keV VM images of each organ at any phases (p < 0.05), except for hepatic parenchyma in EAP. Aortic CNRs in EAP on 65- and 50-keV images of Group B were significantly higher than Group A (p <0.05, respectively). Aortic CNR on 50-keV images of Group A and on 65-keV images of Group B were 11% and 21% higher than 65-keV images of Group A, respectively.

**Conclusions:**

Low-voltage CT simulated by VM image improved contrast enhancement through any phases, while high-concentration contrast material increased only arterial contrast in EAP more effectively.

## Introduction

Many experiments have been conducted to improve the contrast-to-noise ratio (CNR) of abdominal organs for contrast-enhanced computed tomography (CECT) with reduced radiation exposure (Dawson 2004; Fenchel et al. 2004). The administration protocol of iodinated contrast material for dynamic CT, including total iodine dose, iodine concentration, injection rate, and injection duration, have a significant effect on diagnostic performance especially of upper abdominal and vascular diseases, therefore getting so that attainment of higher CNR for organs became of increasing importance (Hänninen et al. 2000; Tsurusaki et al. 2004). The results of numerous investigations consistently demonstrated that administration of higher-concentration contrast materials and a faster bolus injection could lead to higher iodine-dose rate and volumetric rate, causing an increase of effective enhancement and CNR for abdominal organs (Awai et al. 2004; Cademartiri et al. 2005)

Low-tube-voltage CT at 80 or 100 kVp can also improve contrast of vascular and tumor enhancement and applied to abdominal CT, because X-ray generated with low voltage CT is markedly absorbed by high-atomic number substances such as iodinated contrast material (Takahashi et al. 1998; Ravenel et al. 2001; Funama et al. 2005; Nakayama et al. 2005). Recently, fast-switch kVp dual-energy CT using the new technology of fast tube-voltage switching has been developed, with which a virtual monochromatic (VM) image can be reconstructed at any desired monochromatic energy from 40 to 140 kiloelectron voltages (keV) (Wu et al. 2009). Its application makes it possible to create multiple VM images for different voltages reconstructed from one set of dual-energy projection data and to make simulated assessment of those images under the same conditions. Some reports have reported that administration of high-concentration contrast material and low-keV VM image can both improve the CNR for abdominal contrast-enhanced CT, none of them have so far compared the relationship between these two factors under the same conditions (Kulkarni et al. 2012). We hypothesized that the impact of low-voltage CT on contrast enhancement of abdominal organs may be greater than that of high-concentration contrast material during any dynamic phases.

Low-voltage 80 kVp and usual-voltage 120 kVp conventional CT images can be simulated by VM images of two different energies (50 keV and 65 keV) obtained with fast-switch kVp dual-energy CT, respectively (Wu et al. 2009). In this study, we evaluated CNRs for multiphasic contrast-enhanced CT of aorta, portal vein, and hepatic parenchyma by using two contrast materials at different concentrations and VM images for the two-different voltage simulation. The purpose of this study was to compare the effects of high-concentration contrast material and low-voltage CT simulated by VM imaging on contrast enhancement at multiphasic CT of the upper abdomen.

## Materials and methods

### Patient population

Our institutional review board approved this retrospective study and waived the requirement for informed consent. We searched our medical records of our hospital for patients who underwent initial investigation by multiphasic contrast-enhanced CT of the upper abdomen with dual-energy CT scanner using dual-energy technique for detection of known or suspected liver or pancreas tumors between January 2010 and March 2011 for inclusion of this study. We found such 80 patients, but eight patients were excluded from this study because it was difficult to place the region of interest (ROI) accurately on the organ concerned for measurement of attenuation due to total occlusion of the portal vein (n = 1), major tumor invasion of the liver (n = 2), or too thin a subcutaneous fat layer of the anterior abdominal wall (n = 5). As a result, a total of 72 patients were included in this study.

The patients were divided into two groups. Group A consisted of 34 patients who underwent CT examination using the protocol with 300 mgI/ml contrast material (22 men and 12 women; mean age, 65 years; age range, 20–90 years; mean body weight, 60 kg; weight range, 40–86 kg). Group B consisted of 38 patients who underwent CT examination using the protocol with 350 mgI/ml contrast material (21 men and 17 women; mean age, 65 years; age range, 33–84 years; mean body weight, 61 kg; weight range, 40 – 89 kg).

Thirty patients were clinically diagnosed with hepatic cirrhosis (Group A: n = 16; Group B: n = 14), and liver tumors were clinically or histopathologically proven in 36 patients (25 hepatocellular carcinomas, 6 liver metastases, 3 cholangiocarcinomas and 2 combined hepatocellular-cholangiocellular carcinomas). Eighteen patients were diagnosed by biopsy or radical surgery with pancreatic adenocarcinoma and one patient with carcinoma of the ampulla of Vater. None of the patients showed tumor invasion of the major vessels or obstructive jaundice, which could affect contrast enhancement of the abdominal organs and portal systems (Table 1).

**Table 1:** **Patient**-**group demographics**

Patients back ground	Group A	Group B
Number of patients	34	38
Number of men	22	21
Age (y)	65 (20–90)	65 (33–84)
Weight (kg)	60 (40–86)	61 (40–89)
Clinical condition		
Hepatic cirrhosis	16	14
Hepatocellular carcinoma	14	11
Pancreatic adenocarcinoma	7	11
Contrast material infusion protocols		
Administered iodine dose (mgI/kg)	600	600
Iodine concentration (mg/mL)	300	350
Injection duration (s)	30	26
Volumetric rate (mL/sec/kg)	0.067	0.067
Iodine-dose rate (mgI/sec/kg)	20	24

### Dual-energy CT technique

Multiphasic CT was performed with a fast kVp switching 64-section multi-detector row dual-energy CT scanner (Discovery 750 HD; GE healthcare, Milwaukee, WI). This scanner consists of a single-source CT tube that switches rapidly within a fraction of a millisecond between 80 and 140 kVp, so that two complete sets of projection data at 80 and 140 kVp could be collected simultaneously with minimal influence from misregistration artifacts. The tube-current setting was automatically preset at 600 mA by the software for the dual-energy CT system. The field of view covered by the detector was (34.5 – 40.0) cm^2^ depending on the physical size of the patient. Data were acquired with a detector configuration of 64 × 0.625 mm, rotation time of 0.5 second and pitch of 1.375. Collimation of 0.625 × 64 slices was chosen for dual energy acquisition. After scanning, 5 mm-thick, conventional 140-kVp CT images were automatically reconstructed for clinical use. The patient dose indexes of the volume CT dose index (CTDIvol) and the dose-length products were recorded from the console of the CT scanner. The CTDIvol value displayed by the scanner was 12.72 mGy for each scan.

### Contrast material infusion protocols and multiphasic data acquisition

After the acquisition of anteroposterior abdominal scout radiographs, the patients underwent multiphasic dual-energy CT scanning comprising early arterial, late arterial and portal venous phase scans. Conventional abdominal CT scanning before the injection of contrast material were obtained in all patients, and additional scanning of the equilibrium phase were performed when clinically indicated. All patients were intravenously injected with 600 mgI/kg of total body iodine load of non-ionic contrast material by means of insertion of a 20- or 22-gauge IV catheter into an antecubital vein and a power injector (Dual-Shot Type D; Nemoto Kyorindo, Tokyo, Japan). Two-different injection protocols of contrast material were used: an injection of 300-mgI/ml concentration contrast material, iomeprol (Iomeron 300; Bracco-Eisai Co., Ltd., Tokyo, Japan) for 30 seconds for Group A, and an injection of 350-mgI/ml concentration contrast material, iomeprol (Iomeron 350; Bracco-Eisai Co., Ltd.) for 26 seconds for Group B. The volumetric rate of the contrast material per patient body weight was the same for the two groups (0.067 mL/sec/kg for Group A and Group B). On the other hand, the iodine-dose rate per patient body weight was 20 mgI/sec/kg for Group A and 24 mgI/sec/kg for Group B. Scanning delay after the injection was determined with a semi-automatic computer-assisted bolus tracking program (Smart Prep; GE Healthcare), and early arterial, late arterial, and portal venous phasic CT scanning was started 8, 20 and 50 seconds respectively after the trigger (the threshold level set at an increase of 100 HU over the baseline CT number of the abdominal aorta at the level of the celiac axis by 140-kVp CT image on the console).

### Post-processing of dual-energy data

The dual-energy data thus obtained were sent to a computer workstation (Advantage Windows; GE healthcare) and the 5 mm-thick VM image of each phase was reconstructed on the workstation with analytical software for dual-energy data (GSI viewer; GE healthcare). The paired-projection data collected by dual-energy scan were analyzed in terms of the material decomposition process to determine the material density projection after a series of calibrations and corrective steps. A monochromatic energy image could be generated from the weighted sum of material density projections with their corresponding mass attenuation coefficients at a given energy. For any virtual keV between 40 and 140 keV, the object is depicted on the workstation as if imaged with a monochromatic X-ray beam that simulated keV (Wu et al. 2009). VM images of two different energies (50 keV and 65 keV) were generated as the images that had attenuation properties similar to conventional CT images at 80 kVp and 120 kVp, and conventional CT images at 140kVp were also transferred to the workstation as reference images. As a result, a set of nine images was obtained per patient for one examination: 5-mm thick 140-kVp conventional CT, 65-keV VM and 50-keV VM images for each of the early arterial, late arterial and portal venous phase.

### Quantitative analyses

Quantitative measurements were performed at the same workstation by a radiologist with 8 years’ experienced. A total of nine image sets from one patient were displayed side by side on two screens with a preset abdominal window. For each of the image sets, the attenuations of abdominal aorta, portal vein, hepatic parenchyma, paraspinal muscle and subcutaneous fat tissue in the anterior abdominal wall were measured by manually placing the circular or oval ROI cursor on the organ concerned at a slice level which included the hepatic hilum. The shape, size and position of the ROIs were kept constant with the cut-and-paste function of the workstation. The displacement of abdominal organs among the early arterial, late arterial and portal venous scanning attributable to the patient’s continuous breathing caused slight differences in the cursor positions, so that each of the ROIs was carefully confirmed to be placed on the organ of interest and manually adjusted to nearly the same position on all the image sets. Attenuations of each organ were also measured on unenhanced CT.

Attenuation of the abdominal aorta was recorded from a single ROI (mean area, 183 cm^2^; range, 77–260) that was relatively the same size as the vessel lumen at the level of the celiac trunk. Calcifications and soft plaques were carefully avoided for each ROI. Attenuation of the portal vein was also recorded from a single ROI (mean area, 93 cm^2^; range, 40–197) at the level of the main portal vein. ROIs as large as the vessel were carefully placed to reduce the effect of inhomogeneous attenuation of the vessel. Attenuation of the hepatic parenchyma was recorded as the average of the CT values of 3 ROIs (mean area, 400 cm^2^; range, 384–414) placed on the anterior and posterior segment and on the left lobe of the liver. Areas containing hepatic tumors and focal changes in the liver, large vessels and prominent artifacts were also avoided. Attenuation of the paraspinal muscle was recorded from a single ROI (mean area, 196 cm^2^; range, 118–220) without including the macroscopic area of fat infiltration. Image noises were measured for each image set as standard deviations of pixel values from ovoid ROIs (mean area, 201 cm^2^; range, 180 – 209) drawn on the homogenous region of subcutaneous fat tissue in the anterior abdominal wall.

The contrast-to-noise ratio (CNR) for aorta, portal vein and liver parenchyma in comparison with that for muscle was calculated with the equation: CNR = (ROI_o_ – ROI_m_)/SD_n_, where ROI_o_ was the attenuation of a given organ, ROI_m_ was the attenuation of the paraspinal muscle, and SD_n_ was the average standard deviation of subcutaneous fat tissue (or the image noise). In addition, increases in the mean CNRs for 50-keV VM images in Group A, 65-keV VM images in Group B, and 50-keV VM images in Group B over those for the 65-keV VM images in Group A were calculated and defined as ratios of increase in CNR for each phase.

### Qualitative analyses

Image quality of 140-keV conventional CT and 65- and 50 keV VM images was evaluated by two board-certified radiologists with 8 and 21 years of experience in abdominal CT, who were blind to acquisition parameters of each image and clinical background. The images at the anatomical slice level including the hepatic hilum in the early arterial, late arterial, and portal venous phase were selected phase by phase and then randomly serialized on the workstation with the standard abdominal window (window level 50 HU, window width 350 HU). The radiologists independently graded the images for image contrast, image noise and overall image quality. Image contrast and overall image quality of the conventional and VM images were rated on a 4-point scale: 1, unacceptable; 2, acceptable; 3, good; 4, excellent. Image noise as graded similarly: 1, severe noise present and unacceptable; 2, moderate noise present and interfering; 3, mild noise not interfering with depiction of structures; 4, no substantial noise. The initial setting of window level and width was set as the standard abdominal window.

### Statistical analysis

Statistical analysis was performed with JMP software version 9.2 (SAS Institute Inc., Cary, NC). P < 0.05 was considered significant for all statistical analyses. The data for the 140 kVp conventional images were also viewed for reference, but not statistically evaluated.

First, student’s *t* test was used to compare the background factors (patient age, body weight) and attenuation of each organ on unenhanced CT of Groups A and B. Second, intragroup attenuation of aorta, portal vein, liver parenchyma, paraspinal muscle and subcutaneous fat tissue (image noise) for each phase observed on 65 and 50-keV VM images was statistically compared by using the paired *t* test. The same analyses were performed for comparison of CNRs for each organ (aorta, portal vein and liver parenchyma). Third, intergroup CNRs for each organ were statistically compared for Groups A and B by using student’s *t* test for the corresponding images of the 65- and 50-keV VM images at each phase. Finally, the scores for image quality given by the two radiologists to 65- and 50 keV VM images for each group were statistically compared by student’s *t* test.

## Results

### Background-factors evaluation

There were no significant differences in patient age and body weight between Groups A and B. No significant differences were observed in clinical findings of liver cirrhosis. Mean attenuation on unenhanced CT of Groups A and B were 42.7 HU ± 5.1 (mean ± standard deviation) and 43.7 HU ± 4.5 for the abdominal aorta, 35.9 HU ± 7.9 and 37.8 HU ± 7.7 for the portal vein, and 58.1 HU ± 7.6 and 55.7 HU ± 7.4 for the hepatic parenchyma, respectively. There were no significant differences observed in attenuation of each organ on unenhanced CT between Groups A and B (p > 0.05).

### Quantitative analysis

#### Attenuation and Image noise

Mean attenuations for aorta, portal vein, hepatic parenchyma and paraspinal muscle and image noise on 65 and 50-keV VM images together with those for 140-keV conventional images as reference are shown in Table 2. Mean attenuations for each organ observed on 140-kVp conventional, 65-keV and 50-keV VM images increased in that order at any phase for both patient groups. Moreover, mean attenuations on 50-keV VM images were significantly higher than those on 65-keV VM images for any organ at any phase for both groups (p < 0.05, respectively).

**Table 2:** **Mean attenuation values for the aorta**, **portal vein**, **hepatic parenchyma and paraspinal muscle** (**HU**) **and image noise**

		Group A			Group B		
		Attenuation (HU)			Attenuation (HU)		
	Phase	140 kVp	65 keV	50 keV	140 kVp	65 keV	50 keV
Aorta	Early arterial	296 ± 42.2	422 ± 65.6	751 ± 119*	355 ± 56.2	511 ± 85.0	907 ± 156*
	Late arterial	302 ± 54.2	441 ± 82.9	787 ± 151*	282 ± 75.6	398 ± 93.7	705 ± 172*
	Portal venous	154 ± 39.0	207 ± 20.3	368 ± 63.2*	158 ± 22.1	219 ± 23.0	375 ± 32.5*
Portal vein	Early arterial	77.4 ± 22.2	94.8 ± 23.4	152 ± 39.7*	82.0 ± 23.4	107 ± 30.5	175 ± 53.4*
	Late arterial	146 ± 34.5	206 ± 45.1	353 ± 81.6*	170 ± 36.8	250 ± 50.4	436 ± 91.2*
	Portal venous	154 ± 18.9	219 ± 25.3	378 ± 43.0*	163 ± 19.7	233 ± 23.6	398 ± 47.3*
Hepatic	Early arterial	69.7 ± 9.75	74.9 ± 9.09	101 ± 16.8*	69.0 ± 9.25	78.1 ± 10.3	105 ± 18.1*
Parenchyma	Late arterial	89.7 ± 16.4	141 ± 16.8	219 ± 26.3*	91.8 ± 13.6	145 ± 13.2	225 ± 22.9*
	Portal venous	113 ± 10.1	141 ± 14.4	221 ± 25.2*	115 ± 10.3	140 ± 15.0	217 ± 24.9*
Paraspinal	Early arterial	58.2 ± 4.98	64.5 ± 5.07	84.2 ± 8.18*	57.5 ± 4.67	64.8 ± 7.15	85.3 ± 11.9*
Muscle	Late arterial	61.3 ± 7.77	67.9 ± 7.77	89.4 ± 11.1*	68.8 ± 7.10	68.8 ± 7.10	92.0 ± 12.7*
	Portal venous	66.6 ± 6.09	73.9 ± 6.23	101 ± 9.76*	65.0 ± 5.62	74.6 ± 7.25	99.3 ± 18.8*
		Image noise			Image noise		
Image noise	Early arterial	8.27 ± 1.30	8.06 ± 1.81	13.1 ± 1.81*	8.56 ± 1.50	8.42 ± 1.84	13.6 ± 2.03*
	Late arterial	8.52 ± 1.38	8.24 ± 1.64	13.3 ± 2.08*	8.84 ± 1.76	8.91 ± 2.11	13.4 ± 2.23*
	Portal venous	8.57 ± 1.39	8.44 ± 1.57	13.5 ± 1.84*	8.96 ± 1.92	8.61 ± 2.05	13.8 ± 1.91*

Data are shown as mean attenuation ± standard deviation.

*Differences were statistically significant between 65-keV and 50-keV images (p < 0.05).

With regard to image noise, mean image noises on 50-keV VM images were significantly higher than that on 65-keV VM images (p < 0.05, respectively), which was similar to that on 140-kVp conventional images, at any phase for both groups.

### CNR

Mean CNRs for aorta, portal vein and hepatic parenchyma are shown in Table 3. Mean CNRs for each organ observed on 140-kVp conventional, 65-keV and 50-keV VM images increased in that order, making exceptions of hepatic CNRs in the early arterial phase for both groups. Mean CNRs on 50-keV VM images were significantly (p < 0.05) higher than those on 65-keV VM image for each organ at any phase for both patient groups, with the exception of hepatic CNRs in the early arterial phase for group A.

**Table 3:** **Mean CNRs for the aorta**, **portal vein and hepatic parenchyma**

								Intergroup comparison	
		Group A			Group B			Group A vs B	
		CNR			CNR			p	
	Phase	140 kVp	65 keV	50 keV	140 kVp	65 keV	50 keV	65 keV	50 keV
Aorta	Early arterial phase	29.5 ± 7.17	46.6 ± 12.7	51.7 ± 13.1*	36.6 ± 9.92	56.6 ± 18.0	62.4 ± 16.7*	0.015	<0.01
	Late arterial phase	29.1 ± 8.66	47.5 ± 14.2	53.7 ± 14.8*	25.3 ± 10.0	38.4 ± 16.8	44.1 ± 16.0*	0.018	0.01
	Portal venous phase	10.6 ± 5.02	16.4 ± 4.22	20.1 ± 5.74*	10.8 ± 3.96	16.6 ± 5.28	19.6 ± 4.74*	n.s.	n.s.
Portal vein	Early arterial phase	2.41 ± 2.78	3.88 ± 3.02	5.31 ± 3.17*	2.48 ± 3.60	5.49 ± 4.29	6.88 ± 4.20*	n.s.	n.s.
	Late arterial phase	10.3 ± 4.89	19.0 ± 4.73	22.0 ± 4.57*	13.0 ± 5.17	18.9 ± 5.11	21.8 ± 4.77*	n.s.	n.s.
	Portal venous phase	10.6 ± 2.93	17.8 ± 4.59	20.7 ± 4.38*	11.5 ± 3.51	18.1 ± 5.38	21.4 ± 6.31*	n.s.	n.s.
Hepatic parenchyma	Early arterial phase	1.47 ± 1.29	1.35 ± 1.45	1.36 ± 1.48	1.17 ± 1.87	1.66 ± 1.80	1.55 ± 1.94	n.s.	n.s.
	Late arterial phase	3.37 ± 2.05	9.32 ± 3.16	9.94 ± 2.67*	3.70 ± 1.92	8.73 ± 2.61	9.47 ± 2.57*	n.s.	n.s.
	Portal venous phase	5.69 ± 1.60	8.28 ± 2.46	8.96 ± 2.25*	5.97 ± 1.92	7.56 ± 2.77	8.45 ± 2.83*	n.s.	n.s.

Data are shown as mean ± standard deviation.

*Differences were statistically significant between 65-keV and 50-keV images (p < 0.05).

n.s.: not significant.

Intergroup comparison of mean CNRs for groups A and B at the same voltage setting showed significant differences only for the aorta in the early and late arterial phases. Aortic CNRs in the early arterial phase on 65 and 50-keV VM images for Group B were significantly (p = 0.015 and <0.01, respectively) higher than those for Group A. On the contrary, aortic CNRs in the late arterial phase on the 65-keV and 50-keV VM images for Groups B were significantly (p = 0.018 and 0.01, respectively) lower than those for Group A.

### Ratios for increase in CNR

The ratios for increase in CNR compared to 65-keV VM images of Group A are shown in Table 4. The aortic ratios on 50-keV VM images of Group A and 65- or 50-keV VM images of Group B showed 1.11, 1.21 and 1.34 CNR increase in the early arterial phase, respectively.

**Table 4:** **Ratios of increase in CNR on each VM image compared to 65**-**keV VM image with 300-mgI/ml concentration contrast material as baseline CNR**

		Group A	Group B	
	Phase	50 keV	65 keV	50 keV
Aorta	Early arterial	1.11	1.21	1.34
	Late arterial	1.13	0.81	0.93
	Portal venous	1.23	1.01	1.20
Portal vein	Early arterial	1.37	1.41	1.77
	Late arterial	1.16	0.99	1.15
	Portal venous	1.16	1.02	1.20
Hepatic	Early arterial	1.01	1.26	1.14
Parenchyma	Late arterial	1.07	0.94	1.02
	Portal venous	1.08	0.91	1.02

### Qualitative analysis

**Figure 1:**
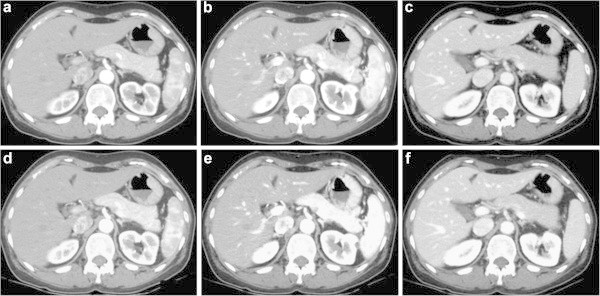
**60-year-old weighed 50 kg and had a normal liver.** **a**, **b**, and **c**: 65-keV VM images; **d**, **e**, and **f**: 50-keV VM images obtained in the early arterial **(a and d)**, late arterial **(b and e)**, and portal venous **(c and f)** phases after administration of 600 mgI/kg of 300-mgI/ml concentration contrast material, which were displayed with the same preset abdominal window (window width, 350 HU; window level, 50 HU). Attenuation of vessels and hepatic parenchyma is increased on the 50-keV VM images compared with the 65-keV VM images.

**Figure 2:**
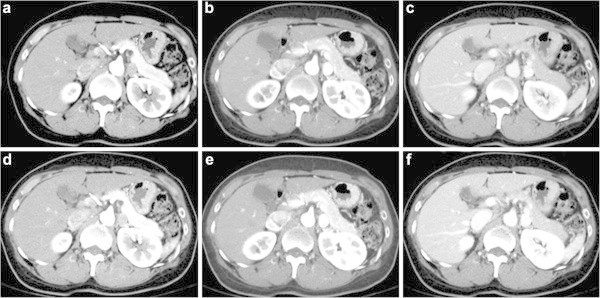
**53-year-old woman weighed 50 kg and had a normal liver.** **a**, **b**, and **c**: 65-keV VM images; **d**, **e**, and **f**: 50-keV VM images obtained in the early arterial **(a and d)**, late arterial **(b and e)**, and portal venous **(c and f)** phases after administration of 600 mgI/kg of 350-mgI/ml concentration contrast material, which were displayed with the standard abdominal window. Attenuation of vessels and hepatic parenchyma is increased on the 50-keV VM images compared with the 65-keV VM images.

Mean scores for evaluation of image quality with respect to image contrast, image noise, or overall image quality are shown in Table 5. The scores for image contrast on 50-keV VM images in the early arterial phase image were noticeably better than those for those on 65-keV VM images for both patient groups, although the scores for image noise and overall image quality were worse (Figures 1 and 2).

**Table 5:** **Mean visual scores for image contrast**, **image noise and overall image quality**

			Group A			Group B		
			Score			Score		
	Phase		140 kVp	65 keV	50 keV	140 kVp	65 keV	50 keV
Image contrast	Early arterial	Reader 1	3.1 ± 0.3	3.4 ± 0.4	3.9 ± 0.2*	3.2 ± 0.4	3.4 ± 0.4	3.9 ± 0.2*
		Reader 2	3.3 ± 0.4	3.5 ± 0.5	3.8 ± 0.3*	3.4 ± 0.5	3.5 ± 0.5	3.8 ± 0.3*
	Late arterial	Reader 1	3.3 ± 0.4	3.8 ± 0.3	3.9 ± 0.2	3.5 ± 0.5	3.9 ± 0.1	4.0 ± 0.0
		Reader 2	3.2 ± 0.5	3.8 ± 0.3	3.9 ± 0.2	3.5 ± 0.5	3.9 ± 0.2	4.0 ± 0.0
	Portal venous	Reader 1	3.4 ± 0.5	3.8 ± 0.3	3.9 ± 0.1	3.4 ± 0.5	3.9 ± 0.1	3.9 ± 0.2
		Reader 2	3.3 ± 0.5	3.8 ± 0.3	4.0 ± 0.0	3.4 ± 0.4	3.8 ± 0.3	3.9 ± 0.1
Image noise	Early arterial	Reader 1	3.7 ± 0.4	3.5 ± 0.4	2.9 ± 0.5*	3.7 ± 0.4	3.4 ± 0.5	2.9 ± 0.4*
		Reader 2	3.9 ± 0.2	3.8 ± 0.7	3.3 ± 0.6*	3.9 ± 0.2	3.6 ± 0.5	3.3 ± 0.7
	Late arterial	Reader 1	3.5 ± 0.5	3.2 ± 0.6	2.7 ± 0.7*	3.6 ± 0.4	3.1 ± 0.7	2.8 ± 0.6
		Reader 2	4.0 ± 0.0	3.7 ± 0.8	3.4 ± 0.8	3.9 ± 0.2	3.8 ± 0.7	3.2 ± 0.9
	Portal venous	Reader 1	3.4 ± 0.5	3.3 ± 0.6	3.2 ± 0.6	3.7 ± 0.4	3.3 ± 0.5	3.2 ± 0.4
		Reader 2	3.9 ± 0.2	3.6 ± 0.4	3.5 ± 0.5	4.0 ± 0.0	3.7 ± 0.4	3.5 ± 0.5
Overall image quality	Early arterial	Reader 1	3.7 ± 0.4	3.4 ± 0.4	3.0 ± 0.3*	3.6 ± 0.4	3.5 ± 0.5	2.9 ± 0.3*
		Reader 2	3.4 ± 0.5	3.5 ± 0.4	3.2 ± 0.6	3.5 ± 0.5	3.2 ± 0.8	3.2 ± 0.8
	Late arterial	Reader 1	3.2 ± 0.4	3.2 ± 0.6	2.9 ± 0.6*	3.5 ± 0.5	3.2 ± 0.6	2.9 ± 0.6*
		Reader 2	3.2 ± 0.5	3.6 ± 0.8	3.2 ± 0.6	3.5 ± 0.5	3.7 ± 0.7	3.2 ± 0.9
	Portal venous	Reader 1	3.5 ± 0.5	3.5 ± 0.5	3.4 ± 0.5	3.7 ± 0.4	3.5 ± 0.5	3.4 ± 0.5
		Reader 2	3.3 ± 0.4	3.5 ± 0.5	3.5 ± 0.5	3.5 ± 0.5	3.7 ± 0.4	3.5 ± 0.5

Data are shown as mean ± standard deviation.

*Differences were statistically significant between 65-keV and 50-keV images (p < 0.05).

n.s.: not significant.

## Discussion

Low-voltage CT of the abdomen has been gaining the attention of investigators because it has the potential to improve contrast enhance for hypervascular liver lesions in the late arterial phase with less contrast material and a lower radiation dose (Nakayama et al. 2005; Altenbernd et al. 2011). It is also reported to be useful for improving enhancement of the pancreas and peripancreatic vasculature or improvement of tumor conspicuity during the pancreatic parenchymal and portal venous phases (Altenbernd et al. 2011; Marin et al. 2010). Moreover, low-voltage CT is used to improve the depiction of anatomically fine structure like peripheral or coronary arteries for the arterial phase and urinary system opacification for excretory phase CT urography (Utsunomiya et al. 2010; Shinagare et al. 2011). Most previous studies of low-voltage CT investigated contrast enhancement during a single phase (Nakayama et al. 2005; Altenbernd et al. 2011; Shinagare et al. 2011) and few studies have investigated contrast for multi-phasic CT. Besides, the use of high-concentration contrast material has been found to yield superior vascular enhancement compared with moderate-concentration contrast material and not to improve contrast enhancement of liver parenchyma on portal phase and late phase images (Awai et al. 2004; Holalkere et al. 2011; Fleischmann 2003). To the best of our knowledge, no studies have compared the effects of high-concentration contrast material and low-voltage CT or low-voltage VM image on contrast for multi-phasic CT of the upper abdomen.

In our study, these effects were evaluated by the simulation using VM images which have become available with the recently developed CT technique of fast-switch kVp dual-energy scanning (Wu et al. 2009). In our institution, dual-energy CT is clinically used for the diagnosis of hepatic and pancreatic tumors based on the clinical utility reported previously, and the radiation dose for dual-energy CT used in this study was almost similar to that for conventional CT (Heye et al. 2012). VM imaging can depict an object as if imaged with a monochromatic beam at any simulated energy from 40 to 140 keV. We employed VM images reconstructed at two different photon energies of 65- and 50-keV on the basis of previously reported findings. The first of these findings was that a comparison between 65-keV VM images and conventional CT images found equivalent performances. In particular, the CNR of VM images at around 65-keV was similar or slightly better to that on conventional 120-kVp CT images (Zhang et al. 2011; Yu et al. 2011). The second, Huda et al. demonstrated that the X-ray tube potentials of 80 and 120 kVp were similar to the representative photon energies of 52 and 66 keV, respectively (Huda et al. 2000). The third was that 120-kVp was the tube voltage in general clinical use for conventional CT, while 80-kVp was commonly used voltage for low-voltage CT of the abdomen (Altenbernd et al. 2011; Marin et al. 2010). Patients had to be scanned twice to compare intra-patient attenuation differences on conventional single-energy CT images at low- and high-tube voltages, while the efficacy of VM image was evaluated by comparing image sets reconstructed at two-different keV from one clinical dual-energy data. It has also been reported that VM imaging improved image quality compared to conventional CT due to a reduction in beam-hardening artifacts (Wu et al. 2009; Matsumoto et al. 2011). A phantom study showed that image noise on VM images for the range of 67–72 keV was significantly lower than that on 120-kVp conventional CT images, with CNR on the VM image highest at 68 keV (Matsumoto et al. 2011). Before starting our study, we considered that those characteristics of VM imaging might make it possible to compare the same clinical data under conditions of better image quality.

Our results demonstrated that low-keV VM images improved aortic and portal venous CNRs in any phases and hepatic CNRs in the late arterial and portal venous phases. In the patient group injected with 300-mgI/ml concentration contrast material, 50-keV VM images showed 11 - 23% increase for the aorta and 16 - 37% for the portal vein in each phase, and 7 - 8% increase in CNR for the liver parenchyma in the late arterial and portal venous phases in comparison with the results of 65-keV VM images. These results indicate that 50-keV VM imaging can improve abdominal CNR in any phase, even when the image niose was increased by the lower keV reconstruction (Lewis et al. 2013). Our results also demonstrated that use of high-concentration contrast material can increase the aortic enhancement significantly in the early arterial phase. As aortic CNRs on 50-keV VM images for Group A and on 65-keV VM image for Group B increased by 11% and 21% over CNR for the 65-keV VM images for Group A respectively, greater improvement in aortic CNR in the early arterial phase could be achieved with the use of high-concentration contrast material compared to the findings for 50-keV VM images. Use of high concentration, which enables a higher iodine delivery rate without increasing injection speed, is thought to be more advantageous than low-voltage CT for CT arteriography in patients with heavy weight, in which image quality deteriorates as a result of increased image noise with low-voltage CT. Moreover, the CNR ratio for the aorta in the early arterial phase on 50-keV VM images for Group B increased by 34% compared to baseline CNR of 65-keV VM images for Group A. These findings suggested that we can choose an appropriate combination of a keV setting and an iodine concentration independently to improve image quality for a specific phase imaging. For example, 1) a combined use of low-keV VM image and high-concentration contrast material can be effective for improving arterial enhancement, and 2) lesion-to-surrounding tissue contrast of either hypervascular or hypovascular tumors would probably be increased with low-keV VM image in the late arterial and portal venous phase.

There have been a few clinical reports focusing on the effectiveness of abdominal VM images reconstructed at low keV and appropriate weighing factor of dual-source dual energy CT (Kulkarni et al. 2012; Maturen et al. 2012; Kim et al. 2010; Sun et al. 2012). Generally, low-voltage CT is supposed to result in deterioration of image quality as a result of increased image noise especially in patients with weighing 70 kg or more because the quantity of penetrating photons must decrease substantially at the same level of radiation exposure (Sun et al. 2012). The increase in image noise associated with low-voltage CT can be partially offset by an increase in the tube-current-time product with a reduction in total radiation. As was expected in our study, noise level on 50-keV VM images was higher than on 65-keV VM images. The results of visual evaluation indicated that image qualities of 50-keV VM images were acceptable, although further study is needed to evaluate the practical utility of low-voltage VM imaging for the abdomen. There were several potential limitations to our study. First, this was a retrospective study with a small number of patients with a variety of backgrounds. A further prospective trial is thus needed to validate our preliminary results. Second, no saline chaser was used after injection of contrast material, although previous studies had demonstrated that a saline chaser was effective to enhance the contrast for abdominal organs and to reduce the total dose of contrast material (Dorio et al. 2003). When a certain volume of high-concentration contrast material was trapped in a capacitance vein, it could be interpreted as more iodine having remained in the dead space. It might be possible to underestimate image contrast when using high-concentration contrast material. Third, simulation of low-voltage CT using VM imaging was utilized in this study. Noise level of low-keV VM image may not be same as that of low-kVp CT with same dose. Therefore, further study using low-kVp CT is necessary. Fourth, we could not evaluate either the visualization of peripheral arteries at CT arteriography, or diagnostic value of hyper- or hypo-vascular tumors because of the low prevalence of malignant tumors in our study population.

To summarize, low-keV VM image improved contrast enhancement for vasculature and hepatic parenchyma in any phases of multi-phasic upper-abdominal CT, while use of high-concentration contrast material improved only arterial contrast in the early arterial phase. A combination of low-keV VM imaging and high-concentration contrast material administration was effective for improving arterial enhancement.
